# Evaluation of protective efficacy induced by virus-like particles containing a *Trichinella spiralis* excretory-secretory (ES) protein in mice

**DOI:** 10.1186/s13071-016-1662-7

**Published:** 2016-07-04

**Authors:** Su-Hwa Lee, Sang-Soo Kim, Dong-Hun Lee, Ah-Ra Kim, Fu-Shi Quan

**Affiliations:** Department of Biomedical Science, Graduate School, Kyung Hee University, Seoul, South Korea; Department of Medical Zoology, Kyung Hee University School of Medicine, Seoul, South Korea

**Keywords:** *Trichinella spiralis*, Virus-like particle, Cholera toxin, Vaccine, Protection

## Abstract

**Background:**

The frequent outbreaks of human trichinellosis globally underscore the need to develop effective vaccine. We hypothesized that a novel vaccine could improve vaccine efficacy against *Trichinella spiralis*.

**Methods:**

In this study, we developed virus-like particles (VLPs) containing the 53 KDa excretory/secretory (ES) protein of *T. spiralis* and the influenza matrix protein 1 (M1) as a core protein, and investigated the protective efficacy of the VLPs alone or with cholera toxin (CT) in a mouse model.

**Results:**

Intramuscular immunization induced *T. spiralis*-specific IgG, IgG1 and IgG2a antibody responses before and after challenge infections in the sera. These antibody responses were significantly enhanced in mice immunized with adjuvanted VLPs. Upon challenge infection, vaccinated mice showed significantly reduced worm burden in the diaphragm. Protective immune responses and efficacy of protection were significantly improved by immunization with VLPs together with CT adjuvant.

**Conclusions:**

Our results are informative for a better understanding of the protective immunity induced by *T. spiralis* VLPs, and will provide insight into designing safe and effective vaccines.

**Electronic supplementary material:**

The online version of this article (doi:10.1186/s13071-016-1662-7) contains supplementary material, which is available to authorized users.

## Background

Trichinellosis is a parasitic infection caused by *Trichinella spiralis*, which is a serious parasitic zoonosis and a globally endemic disease [1–3]. Human infection is commonly the result of eating raw or undercooked meat containing *Trichinella* larvae. Pork and its products are closely associated with outbreaks of human trichinellosis. The global prevalence of trichinellosis is difficult to evaluate, but as many as 11 million people may be infected. The frequent outbreaks of human trichinellosis globally underscore the need to develop an effective vaccine [4–6]. The development of vaccines would have significant impact towards the ultimate goal of disease elimination [7, 8].

Natural parasite extracts, recombinant protein, synthetic peptides, attenuated phage display and genetic immunization have been used for vaccine studies. Radiation, ultraviolet-attenuated or DNA-plasmid vaccines for *Trichinella spiralis* were found to be highly protective in experimental animals. However, such vaccines are not well suited for field use [5, 6, 9–11].

Recombinant vaccines based on virus-like particles (VLPs) or nanoparticles have displayed promising safety and efficacy in preclinical and clinical studies [12]. Virus-like particles resemble viruses, but do not contain any viral genetic material. Thus, they do not replicate, having advantages for safety [13–15]. VLPs contain repetitive high density displays of viral surface proteins, which present conformational epitopes that can elicit strong cellular and humoral immune responses [12].

Immunization with *T. spiralis* ES protein elicits a robust immune response, and resulted in complete protection against infective larvae [16]. The 53 kDa protein of *T. spiralis* has been used as an immunomodulatory protein for treating inflammatory disease such as bowel diseases [17]. The *T. spiralis* 53 kDa protein is reported to be a novel serological marker and vaccine candidate [18]. The 53-kDa recombinant proteins provide early and species-specific antibody responses in mice infected with *T. spiralis* [19]. However, there is no report on vaccine efficacy of *T. spiralis* 53 kDa against challenge infection. Thus, we were interested to test the hypothesis that *T. spiralis* 53 kDa protein in virus-like nanoparticle form could be an important immunogen which could induce humoral and/or cellular immunity.

To the best of our knowledge, in this study for the first time VLPs derived from parasite *T. spiralis* were produced. These novel VLPs containing *T. spiralis* 53 kDa protein and influenza matrix M1 as a core protein were evaluated as a potential vaccine. We also investigated the effect of CT as an adjuvant for the VLP vaccine.

## Methods

### Parasite, virus, cells and antibodies

Korean isolate of *T. spiralis* was obtained from specific pathogen-free female, inbred Sprague–Dawley (SD) rats, aged 8 weeks, maintained by serial oral passage. Influenza virus (A/PR/8/34) was used to infect MDCK cells. *Spodoptera frugiperda* Sf9 cells were maintained in suspension in serum-free SF900II medium (Invitrogen, Carlsbad, USA) at 27 °C in spinner flasks at a speed of 130 to 140 rpm. Horseradish peroxidase (HRP)-conjugated goat anti-mouse immunoglobulin A (IgA) and G (IgG), IgG1 and IgG2a were purchased from Southern Biotech (Birmingham, USA).

### Preparation of *T. spiralis* antigen

*Trichinella spiralis* excretory/secretory (ES) product was produced as described previously [20–22]. Larvae of *T. spiralis* were isolated from rat muscle tissue by artificial digestion and washed. Clean larvae were incubated in a CO_2_ incubator for 24 h at 37 °C in Petri dish containing RPMI-1640 culture medium without FBS. The culture supernatants were collected by centrifugation. The supernatant was dialyzed and lyophilized. The protein concentration was determined by QuantiPro BCA Assay Kit (Sigma-Aldrich, St Louis, USA). *Trichinella spiralis* ES products were identified by SDS-PAGE (Additional file 1: Figure S1) and stored at -70 °C until use.

### Construction of rBV expressing *T. spiralis* (T653K) and influenza M1

Total RNA was extracted from the *T. spiralis* larvae using RNeasy Mini Kit (Qiagen, Valencia, USA). The RNA was reverse transcribed to cDNA using the Prime Script 1st strand cDNA synthesis kit according to the manufacturer’s instructions (Takara, Otsu, Japan). The cDNA was used as a template to amplify the complete coding sequence of T653K by polymerase chain reaction (PCR). The primers were designed according to the nucleotide sequence of T653K in GenBank (accession number: DQ399914): forward (5′-AAA GAA TTC ACC ATG TTC AGC ATC ACA TTA AA-3′) and reverse (5′-TTA CTC GAG TTA GAA CAA CAA CTG TAG T-3′) with EcoRI and XhoI restriction enzyme sites. The PCR product was inserted into the pFastBac vector (Invitrogen, Carlsbad, USA). For M1 gene cloning, A/PR/8/34 virus was inoculated into MDCK cells and total viral RNA was extracted using an RNeasy Mini kit (Qiagen, Valencia, USA). Reverse transcription (RT) and PCR were performed on extracted viral RNA using the One-Step RT-PCR system (Invitrogen, Carlsbad, USA) with gene specific oligonucleotide primers. The following primer pairs were used forward, M1 (5′- TCC CCC GGG CCA CCA TGA GCC TTC TGA CCG AGG TC -3′); reverse, M1 (5′- TTA CTT CTA GAT TAC TTG AAC CGT TGC ATC TG -3′) with SmaI and XbaI restriction enzyme sites. Following RT-PCR, a cDNA fragment containing the M1 gene was cloned into the pFastBac vector (Invitrogen, Carlsbad, USA). The recombinant plasmid was transformed into *E. coli* DH5-alpha and transferred into a DH10-Bac. The nucleotide sequences of M1 (accession number: EF467824) and T653K (accession number; DQ399914.1) genes in the pFastBac vector were confirmed by DNA sequencing.

### Production of recombinant baculovirus and VLPs

Transfections of DNA containing the above genes were accomplished using cellfectin II (Invitrogen, Carlsbad, USA) with Sf9 cells as recommended by the manufacturer, followed by transformation of pFastBac containing T653K or influenza M1 with white/blue screening. The rBVs were derived by using a Bac-to-Bac expression system (Invitrogen, Carlsbad, USA). To produce VLPs containing T653K and M1, Sf9 cells were co-infected with rBVs expressing T653Kand M1. Cell culture supernatants were collected on day 2 or 3 post-infection, cleared by centrifugation at 6,000 rpm for 30 min at 4 °C to remove cells. VLPs in the supernatants were pelleted by high-speed centrifugation (45,000× *g* for 30 min). The sedimented particles were resuspended in phosphate-buffered saline (PBS) at 4 °C overnight and further purified through a 20–30–60 % discontinuous sucrose gradient at 45,000× *g* for 1 h at 4 °C. The VLP bands were collected and pelleted by high-speed centrifugation (45,000× *g* for 30 min). VLPs were resuspended in 500 μl phosphate-buffered saline (PBS) overnight at 4 °C and protein concentration was determined using a QuantiPro BCA Assay Kit (Sigma-Aldrich, St Louis, USA).

### Characterization of VLPs

VLPs were characterized by Western blots and electron microscopy. For Western blot analysis, mouse serum was used to probe T653K protein. Serum was from BALB/c mice infected with the *T. spiralis* Korean isolate. Serum was collected at week 4 after infection. Monoclonal mouse anti-M1 antibody was used to determine M1 protein content. For electron microscopy, negative staining of VLPs was performed followed by transmission electron microscopy (Tecnai G2 spirit), (FEI, Hillsboro, USA).

### VLP immunization schedule and challenge infection

Female, BALB/c mice (6–8 week-old) were divided into 4 groups; naïve control, *T. spiralis* infection control (TS control), T653K VLP and T653K VLP/CT. Mice were intramuscularly immunized with T653K VLPs with or without CT (25 μg VLPs/mouse, 2 μg CT/mouse, 10 mice in each group). All experimental groups were vaccinated at weeks 0 and 4. Four weeks after the last immunization, 10 mice of each group were challenged orally with 100 *T. spiralis* (Korea isolate) larvae per mouse. Mice were sacrificed 6 weeks after challenge infection, larvae were collected from the mouse diaphragm and counted after the diaphragm was digested with artificial digestion solution (1 % HCl - 1.5 % pepsin). The rate of reduction in larval burden was calculated according to the recovered larvae per gram muscle. Blood was collected from the retro-orbital plexus on week 0, 1, 5, 8 before challenge and on week 1, 4 and 6 after challenge infection. Sera were separated and stored at -20 °C until analyzed for specific antibodies. All animal experiments and husbandry involved in these studies were conducted under the guidelines of the Kyung Hee University IACUC. Kyung Hee IACUC operates under the National Veterinary Research and Quarantine Service (NVRQS) and regulations of the World Organization for Animal Health (WOAH).

### Evaluation of humoral immune responses

Sera from experimental mice were used in an enzyme-linked immunosorbent assay (ELISA) to measure the levels of IgG, IgG1 and IgG2a against *T. spiralis* antigen. 96 well flat-immunoplate (SPL) were coated overnight at 4 °C with 100 μl of *T. spiralis* antigen at a concentration of 4 μg/ml in 0.05 M carbonate bicarbonate buffer (pH 9.6) per well. Serum samples diluted (1:100) in PBST (100 μl/well) were then added in duplicate.

### Cytokine analysis

Individual mouse spleens were collected 2.5 months after immunization from the immunized and naïve groups. Single-cell suspensions were prepared from each spleen. Cells were incubated in 96-well flat culture plates for 2 days at 37 °C in the presence of 5 % CO_2_. Cells in 100 μl of RPMI-1640 were stimulated with 100 μl of 2 μg/ml *T. spiralis* ES Ag. For the cytokine assay, supernatants of spleen cell cultures were collected from each well by separation and stored at -20 °C until use. OptEIA sets (BD Bioscience, San Jose, CA, USA) were used to determine the concentration of interferon-gamma (IFN-γ), interleukin (IL)-2, IL-4 and IL-10 in culture supernatants following the manufacturer’s procedures.

### Statistics

All parameters were recorded for individuals within all groups. Statistical comparisons of data were carried out using the Kruskal-Wallis test and Paired t-test of PC-SAS 9.3. A *P*-value < 0.05 was considered to be significant.

## Results

### Generation of constructs

**Fig. 1:**
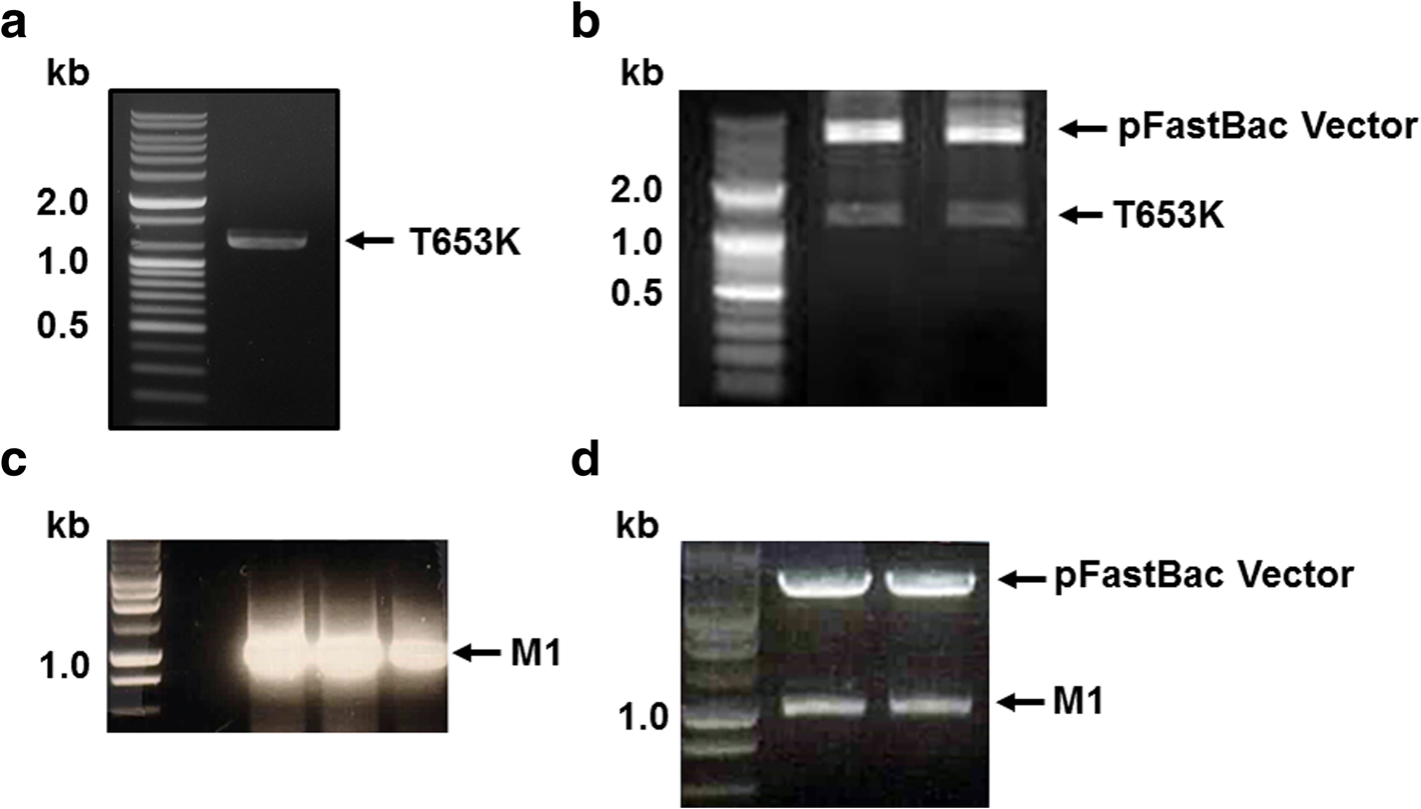
PCR identification of T653K and M1 genes and recombinant plasmids pFastBac-T653K and pFastBac-M1 digested. The *T. spiralis* T653K gene was PCR-amplified from cDNA synthesized using a Prime Script 1^st^ Strain cDNA Synthesis Kit using total RNA extracted from *T. spiralis* Korean isotype (**a**). Influenza M1 gene was PCR amplified fromtotal RNA extracted from influenza virus (A/PR/8/34) (**c**). The *T. spiralis* T653K gene and influenza M1 gene were cloned into pFastBac with EcoRI/XhoI and SamI/XbaI enzymes, respectively, resulting in T653K plasmid (**b**) and M1 plasmid (**d**). Marker: DNA marker; size of T653K: 1,239 bp; size of M1: 1,027 bp

The T653K gene from *T. spiralis* Korean isotype was amplified by PCR and the influenza M1 gene was amplified by RT-PCR with primers containing restriction enzyme sites (Fig. 1a, c). Genes were cloned into pFastBac vectors, and insertion of T653K and M1 in pFastBac expressing vectors was confirmed by cutting with restriction enzyme sites, T653K: BamHI and XbaI, M1: SmaI and XbaI (Fig. 1b, d). The nucleotide sequences of the T653K and M1 genes were found to be identical to the previously published sequences by DNA sequencing.

### Production of VLPs

**Fig. 2:**
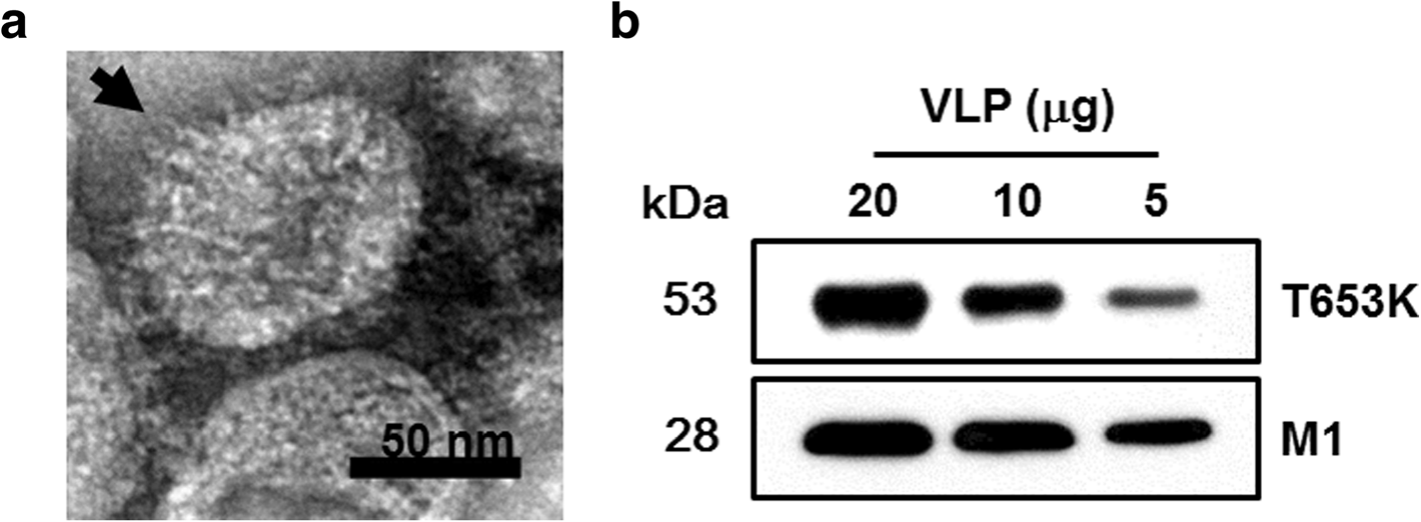
Characterization of virus-like particles (VLPs). Electron microscopy and VLP size determination. Negative staining of VLPs was performed followed by transmission electron microscopy (TEM). The size is between 40 and 120 nm (**a**). Western blot analysis. VLPs (20, 10, 5 μg) were loaded for SDS-PAGE. Polyclonal mouse anti-*T. spiralis* antibody was used to probe T653k protein and anti-M1 monoclonal antibody was used to determine M1 protein (**b**)

VLPs containing influenza T653K and M1 were produced as described in Methods. VLPs were harvested from the culture supernatants of Sf9 cells co-infected with two individual rBVs that express either T653K or M1. The size and morphology of VLPs containing T653K and M1 were examined by electron microscopy. The morphology of VLPs resembles the shape of influenza virus with spikes on their surfaces (Fig. 2a). The particle sizes ranged from approximately 40 to 120 nm. These results show that VLPs expressing T653K and M1 were generated. The incorporation of T653K and M1 into VLPs was confirmed by western blot using antibodies described in the methods (Fig. 2b).

### Humoral immune responses induced by immunization with T653k VLPs

**Fig. 3:**
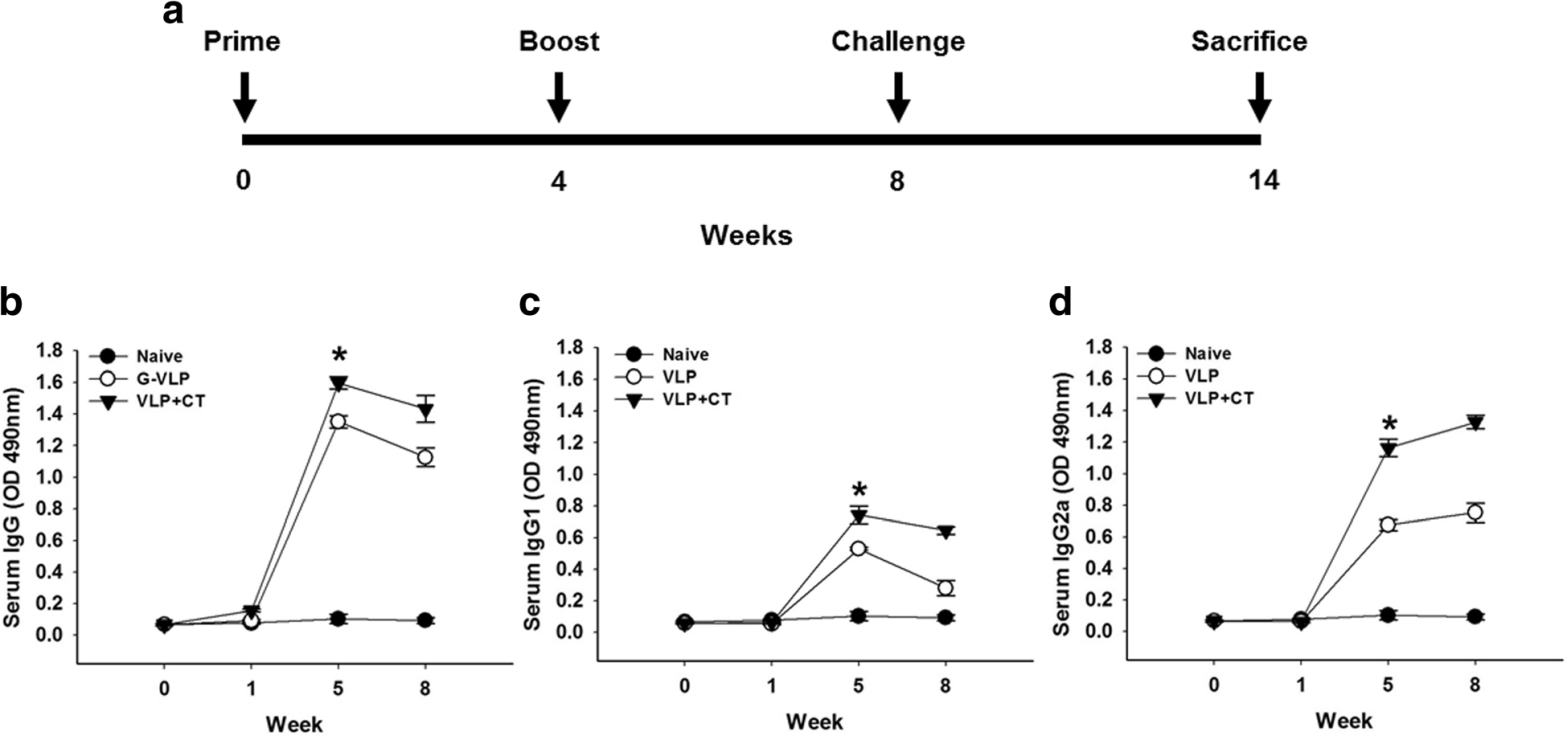
Experimental schedule and *T. spiralis-*specific IgG subtype (IgG1 and IgG2a) responses upon immunization. Mice were immunized twice with VLPs as indicated with a 4-week interval. Challenge infection was performed at week 4 after last immunization (**a**). Enzyme-linked immunosorbent assay (ELISA) plates were coated with *T. spiralis* antigen, as indicated in Methods. *T. spiralis*-specific IgG, IgG1 and IgG2a antibody responses in the sera were determined after prime and boost. Significant differences were found between group VLPs alone and group VLP with cholera toxin (CT) (**b**, **c** and **d**, *P < 0.05)

Mouse sera collected at different time points after immunization were used to measure levels of specific anti-*T. spiralis* IgG and subtype (IgG1 and IgG2a) antibodies (Fig. 3a). Higher levels of *T. spiralis-*specific IgG were found in mice immunized with T653K VLPs with or without CT compared to naïve controls on week 0, 1, 5 and 8, where T653K VLPs with CT showed better *T. spiralis-*specific IgG response than T653K VLP without CT, indicating CT was an effective adjuvant (Fig. 3b; *χ*^2^ = 7.2, *df* = 2, *P* = 0.0273). Higher levels of *T. spiralis-*specific IgG1 and IgG2a antibody responses were also detected in mice immunized with T653K VLPs with CT on week 0, 1, 5 and 8 after immunization compared to VLPs without CT. Higher level of *T. spiralis*-specific IgG2a was found than *T. spiralis*-specific IgG1, indicating that the IgG2a response was dominant (Fig. 3c, d; IgG1: *χ*^2^ = 6.4889, *df* = 2, *P* = 0.039; IgG2a: *χ*^2^ = 7.2, *df* = 2, *P* = 0.0273). Although the IgG2a antibody response was dominant, *T. spiralis*-specific IgG1 antibody was also observed (Fig. 3c), indicating that T653K VLPs vaccination elicited Th1/Th2 mixed immune responses (Fig. 3c, d).

### *Trichinella spiralis*-specific antibody response after challenge infection

**Fig. 4:**
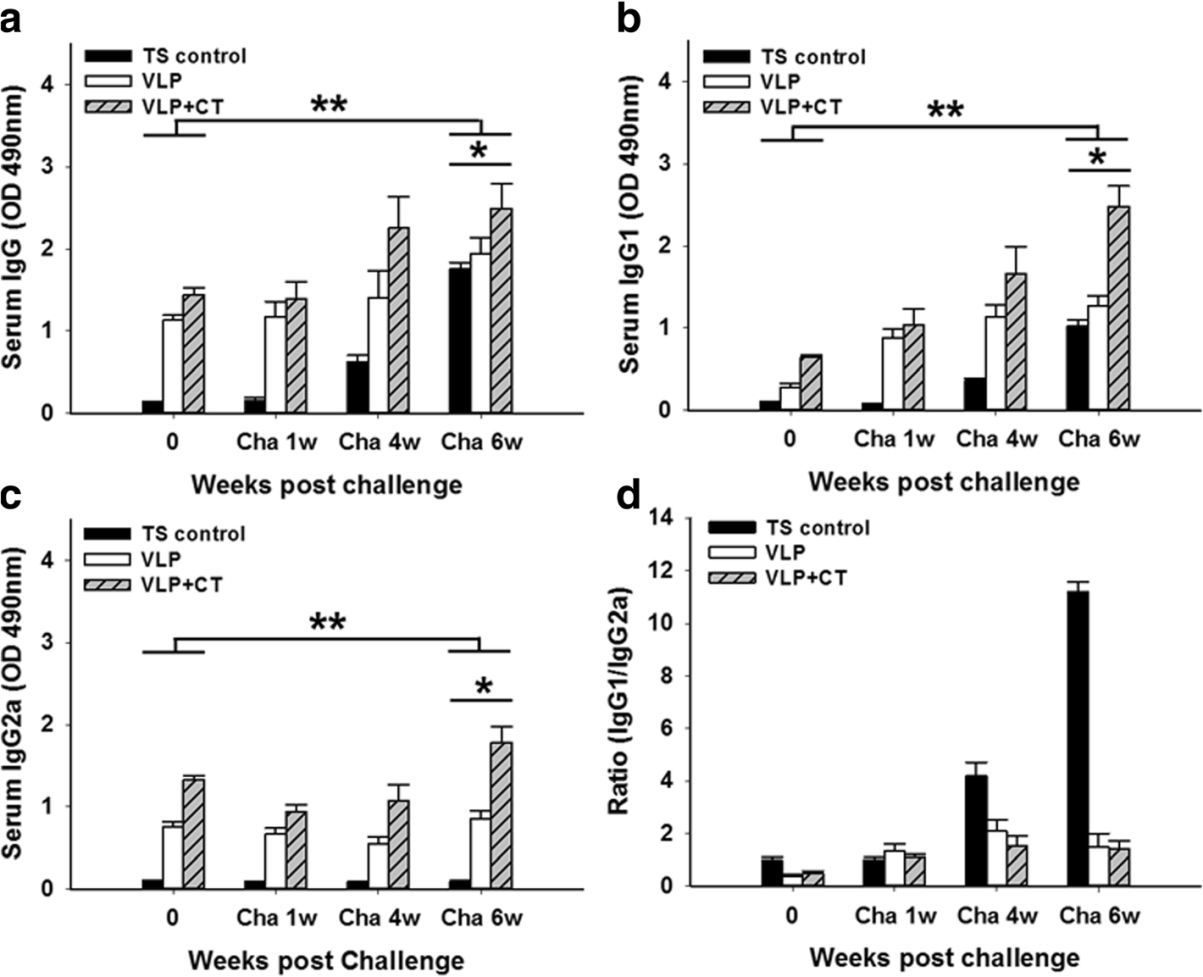
*Trichinella spiralis-*specific antibody responses upon challenge infection. Immunized mice were challenge-infected orally with *T. spiralis* Korean isotype at week 4 after boost and *T. spiralis-*specific IgG, IgG1 and IgG2a antibody responses (**a**, **b** and **c**, *P < 0.05; **P < 0.01) and the ratio of IgG1 to IgG2a in the sera were determined (**d**)

To determine antibody response profiles in serum upon challenge infections, groups of mice were orally challenge infected with *T. spiralis* Korean isolate at week 4 after boost. To better understand the protective immune responses induced by T653K VLPs vaccination after challenge infection, *T. spiralis*-specific IgG, IgG1 and IgG2a were determined in the sera at weeks 1, 4 and 6 after challenge infection (Fig. 4a, b, c). IgG, IgG1 and IgG2a antibody isotypes were significantly increased in groups of mice that received vaccination compared to those in TS control mice (*P* < 0.05 and *P* < 0.01, respectively), in which VLPs vaccination with CT showed higher IgG, IgG1 and IgG2a responses. Significant differences in IgG, IgG1 and IgG2a levels among groups at week 6 post-challenge were found (IgG: *χ*^2^ = 7.2, *df* = 2, *P* = 0.0273; IgG1: *χ*^2^ = 7.2, *df* = 2, *P* = 0.0273; IgG2a: *χ*^2^ = 6.4889, *df* = 2, *P* = 0.039). Significant differences between before and after challenges (week 6 post-challenge) were as follows: IgG: TS control: *t*_(44)_ = 39.99, *P* = 0.0006; VLP: *t*_(44)_ = 16.95, P = 0.0035; VLP + CT: *t*_(44)_ = 27.97, *P* = 0.0013; IgG1: TS control: *t*_(44)_ = 25.7, *P* = 0.0015; VLP: *t*_(44)_ = 21.49, *P* = 0.0022; VLP + CT: *t*_(44)_ = 14.25, *P* = 0.0049, and IgG2a: TS control: *t*_(44)_ = 1.08, *P* = 0.3922; VLP: *t*_(44)_ = 5.48, *P* = 0.0317; VLP + CT: *t*_(44)_ = 5.09, *P* = 0.0364. Importantly, IgG, IgG1 and IgG2a antibody levels were also found to be significantly higher in sera post-challenge than those before challenge, indicating the establishment of infection of larvae in the muscle that induces higher immune response. Especially, IgG on week 4 and 6, IgG1 on week 1, 4 and 6, and IgG2a on week 6 were found to be significantly higher (Fig. 4a, b, c). The VLPs with CT adjuvant showed increased antibody responses compared to that without CT. Interestingly, upon challenge, mice immunized with T653k VLPs with CT showed IgG1-dominant responses (Fig. 4d). This suggests that vaccination with T653K VLPs with CT elicited an IgG1-dominant Th1/Th2-mixed immune responses.

### Protective immunity

**Fig. 5:**
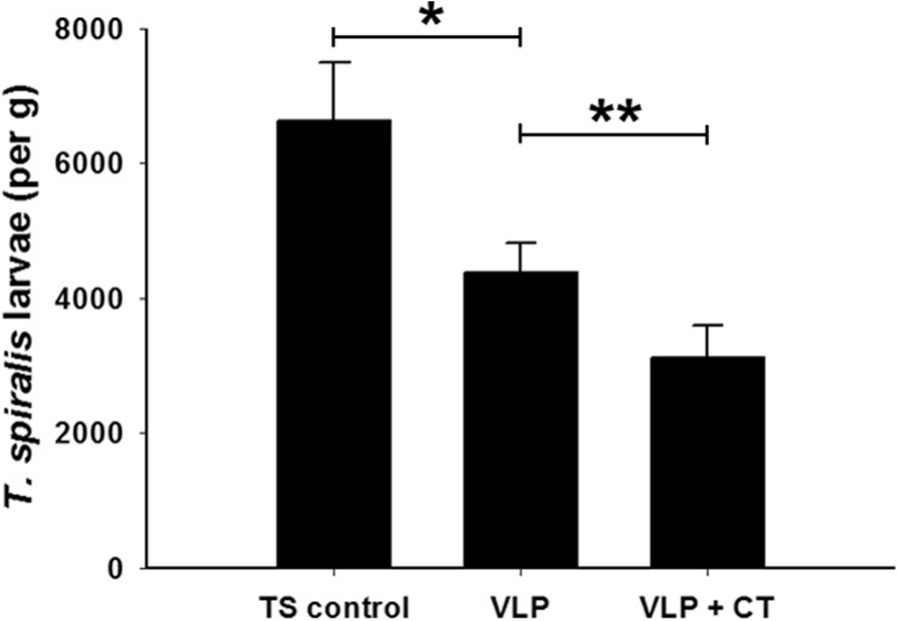
Protection induced by vaccination. The larvae (per gram) from diaphragm were recovered from vaccinated mice after oral challenge infection with 100 *T. spiralis* larvae. Asterisks indicate statistically significant differences (*P < 0.05; **P < 0.01) in worm recovery of the immunized groups compared to TS control group

To determine the efficacies of T653K VLP vaccine, groups of mice including naïve control and vaccinated mice were challenged with 100 larvae of *T. spiralis* at week 8 after vaccination. Mice were sacrificed at week 6 post-challenge and larvae were collected from the diaphragm (Fig. 3a). As shown in Fig. 5, the mice immunized with T653K VLPs with CT showed 53.2 % of larval reduction, and the mice immunized with T653K VLPs showed 34 % of worm reduction, respectively (Fig. 5, *t*_(44)_ = 4.46, *P* = 0.021 and *t*_(44)_ = 3.29, *P* = 0.0462, respectively). The results demonstrated that the intramuscular immunization with T653K VLPs vaccine induced partial protection against challenge infection with *T. spiralis* Korean isolate larvae, and VLPs with adjuvant CT showed better protection.

### Cytokine responses

**Fig. 6:**
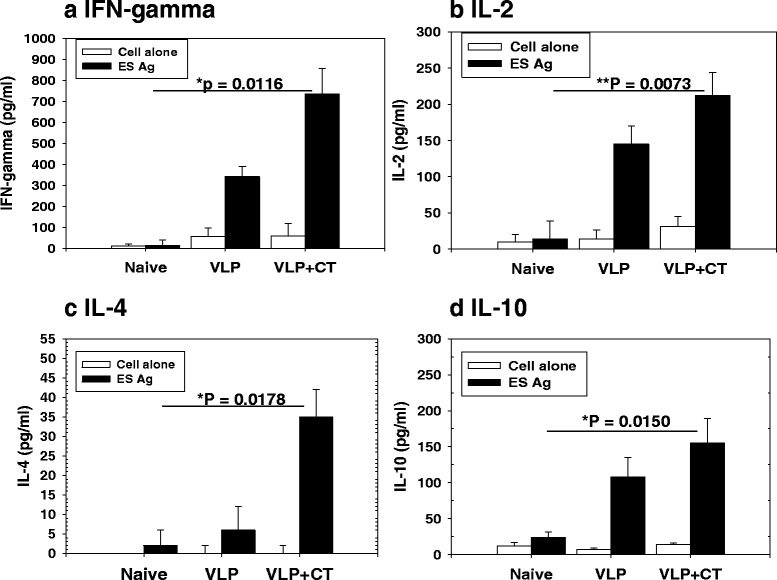
Cytokine responses. The levels of IFN-γ (**a**), IL-2 (**b**), IL-4 (**c**) and IL-10 (**d**) from cytokine-secreting cells after immunization were determined. Significantly higher levels of IFN-γ, IL-2, IL-4 and IL-10 cytokines were produced IN VLP + CT and VLP alone groups compared to naïve controls (*P < 0.05, **P < 0.01)

The levels of IFN-γ, IL-2, IL-4 and IL-10 from cytokine-secreting cells after immunization were determined as indicated in Fig. 6. Significantly higher levels of IFN-γ (*χ*^2^ = 8.9091, *df* = 2, *P* = 0.0116), IL-2 (*χ*^2^ = 9.8462, *df* = 2, *P* = 0.0073), IL-4 (*χ*^2^ = 8.0563, *df* = 2, *P* = 0.0178) and IL-10 (*χ*^2^ = 8.4049, *df* = 2, *P* = 0.0150) cytokines were produced in VLP + CT and VLP groups following *T. spiralis* antigen stimulation compared to naïve controls, in which VLP + CT showed higher levels of cytokines compared to a VLP alone group. Since higher levels of Th1/Th2-like cytokines were found in VLP or VLP + CT groups, we concluded that Th1/Th2-mixed type of immune responses was induced (Fig. 6; *P* < 0.05).

## Discussion

A VLP-induced protective immune response is different from DNA vaccine or protein vaccine. VLP vaccines are genetically engineered and produced in cell cultures. VLP vaccines contain multiple copies of protein antigens, inducing strong humoral and cellular immune responses [12]. DNA vaccines present endogenously expressed antigens to the immune systems, showing relatively low immunogenicity [23]. Recombinant protein vaccines consist of protein antigens produced in heterologous expression systems, inducing antibody response. Compared to VLP vaccines, DNA vaccine mainly induces cellular immunity. F DNA vaccine derived from respiratory syncytial virus (RSV) was not able to induce detectable levels of antibody responses [23]. Compared to VLP vaccines, recombinant H5 HA vaccine is less immunogenic, and vaccination even with 5-fold higher dose did not induce protective immunity [24]. Overall, VLPs offer many advantages in safety, immunogenicity and antigen stability. Taken together, VLP vaccines are promising vaccine candidates against different pathogens.

Virus-like particles (VLPs) represent one of the most exciting new vaccine technologies. VLPs do not contain any viral genetic material, and they have significant potential to elicit a strong immune response, without doing any actual harm [25, 26]. The early vaccines (live, inactivated, subunit) are no longer considered as the most appropriate for new vaccine development [26]. Thus in our current study, for the first time, we used baculovirus expression/VLP technology in a parasite nematode to produce parasite VLPs containing *T. spiralis* T653K and influenza M1 proteins. Our findings provide evidence that T653K VLPs with cholera toxin (CT) or without CT can induce protective immunity against live *T. spiralis* larval infection. The adjuvanted VLPs vaccine significantly improved humoral responses and protection upon challenge infection compared to VLPs alone.

*Trichinella spiralis* excretory-secretory (ES) protein has been demonstrated to induce protective immunity against *T. spiralis* infection. Immunization with *T. spiralis* ES protein elicited *T. spiralis*-specific IgG, IgG1 and IgG2a antibody responses [16]. Vaccination with ES protein microencapsulated in methacrylic acid copolymers elevated the antigen-specific serum IgG1 and IgA antibody responses, inducing Th1/Th2 immune responses that are protective [27]. Recombinant TspSP-1.2 protein derived from *T. spiralis* serine protease gene and Ts-ES-1 (20 kDa) secreted by *T. spiralis* induced partial protections, considering it is a potential candidate for vaccine development against *T. spiralis* infection [28, 29]. These recombinant proteins were produced from an *E. coli* expression system, and vaccine efficacy was not successful. In the current study, we used T653K derived from *T. spiralis* ES Ag 53 kDa as a surface protein of VLPs, for which vaccine efficacy has not been studied previously. Our study indicated that vaccination with *T. spiralis* T653K containing VLPs elicited *T. spiralis*-specific IgG, IgG1 and IgG2a antibody responses and partial protection against *T. spiralis* challenge in mice.

Results shown in the present study indicate that the pattern of IgG1 and IgG2a immune responses is affected by challenge infection. T653K VLPs vaccination induced higher level of *T. spiralis*-specific IgG2a antibodies before challenge and subsequent challenge infection significantly increased the levels of IgG1 antibodies (Figs. 3, 4). An effective vaccine must direct T-helper cells toward the development of Th1, rather than Th2 responses. Moreover, a humoral response is necessary because specific antibodies limit the multiplication of parasite by killing extracellular parasite, either by activating the complement system or by opsonizing the parasites for phagocytosis and macrophage killing [30–32]. Importantly, in the present study, IgG1 antibodies before challenge and IgG2a antibody after challenge infection were also induced, indicating a Th1/Th2-mixed type of immune response, which is important for protective immunity against *T. spiralis* infection [33, 34]. Induction of IgG1 antibody responses upon challenge might be necessary since parasite-specific antibodies and the associated Th2 responses have been reported to limit worm establishment and may even play a role in diminishing the effect of challenge infections [35]. Further studies are needed for better understanding of the immune mechanisms affecting the pattern of antibody isotypes induced by vaccination.

Cholera toxin (CT) is known to be a potent adjuvant. CT can induce maturation of dendritic cells and augment the priming of CD4^+^ T cells and the antigen presentation by dendritic cells and B cells [36–39]. However, the role of CT adjuvant in parasite vaccine fields is unknown. In the present study, for the first time, we investigated protective efficacy in mice immunized with VLPs alone or with CT-adjuvanted VLPs. Cholera toxin (CT) has been widely shown to be effective as a potent mucosal vaccine adjuvant. There is a strong supporting report, in which cholera toxin by nonconventional adjuvant pathway induces protective memory responses after epicutaneous vaccination [40]. In the present study, mice intramuscularly immunized with CT-adjuvanted VLPs showed significantly enhanced T653K VLP-induced *T. spiralis*-specific immune response. All immunized mice were partially protected against challenge infection with *T. spiralis*, in which mice immunized with CT-adjuvanted VLPs showed significant decrease in worm burden compared to mice immunized with VLPs alone (Fig. 5), indicating that the adjuvant plays an important role in enhancing the protective efficacy. Mice immunized with CT-adjuvanted VLPs showed significantly improved protection, indicating the use of a safe and effective adjuvant would have a significant impact for developing VLP vaccines. Since a careful selection of adjuvant is important depending on specific vaccine antigens and desired types of protective immunity, further studies are needed for better understanding of the immune mechanisms induced by adjuvants [41].

## Conclusions

Overall, the present studies provide new insight into *T. spiralis* VLPs-induced protective efficacy. Intramuscular immunization with VLPs alone or with CT elicited a systemic Th1/Th2-mixed type of immune response and produced a partial protection against *T. spiralis* infection in mice. CT-adjuvanted VLPs showed significantly increased immunogenicity.

## Abbreviations

CT, cholera toxin; ELISA, enzyme-linked immunosorbent assay; ES, excretory/secretory; HRP, Horseradish peroxidase; PBS, phosphate-buffered saline; SD’, Sprague–Dawley; SDS-PAGE, sodium dodecyl sulphate-polyacrylamide gel electrophoresis; VLP, virus-like particle.

## Additional file

Additional file 1: Figure S1.
*Trichinella spiralis* ES product was separated by sodium dodecyl sulphate-polyacrylamide gel electrophoresis (SDS-PAGE) in 12 % polyacrylamide gels using a Mini-PROTEAN Tetra Cell electrophoresis unit (Bio-Rad, USA). *Trichinella spiralis* ES product (40, 20, 10, 5 μg) was loaded and incubated at 150 V for 1 h. To determine the proteins in *T. spiralis* ES product, the gel was stained with coomassie blue. *Trichinella spiralis* T653k protein was detected in *T. spiralis* ES product. (PDF 45 KB)
