# Supplementary figures and images for: Evaluation of protective efficacy induced by virus-like particles containing a Trichinella spiralis excretory-secretory (ES) protein in mice
Source: Parasit Vectors. 2016 Jul 4;9:384. doi: 10.1186/s13071-016-1662-7 (PMC4932752; doi:10.1186/s13071-016-1662-7)

Figure S1

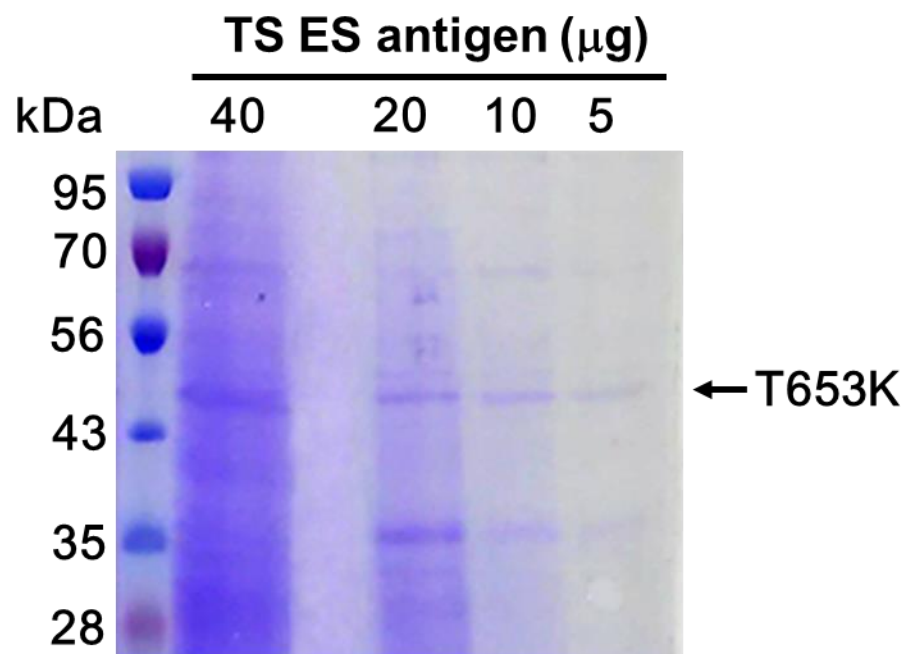

Supplement: Additional file 1: Figure S1. — Trichinella spiralis ES product was separated by sodium dodecyl sulphate-polyacrylamide gel electrophoresis (SDS-PAGE) in 12 % polyacrylamide gels using a Mini-PROTEAN Tetra Cell electrophoresis unit (Bio-Rad, USA). Trichinella spiralis ES product (40, 20, 10, 5 μg) was loaded and incubated at 150 V for 1 h. To determine the proteins in T. spiralis ES product, the gel was stained with coomassie blue. Trichinella spiralis T653k protein was detected in T. spiralis ES product. (PDF 45 KB) [file 13071_2016_1662_MOESM1_ESM.pdf]
